# Cellular Oxidative Stress in Pediatric Leukemia and Lymphoma Patients Undergoing Treatment Is Associated with Protein Consumption

**DOI:** 10.3390/nu12010075

**Published:** 2019-12-27

**Authors:** Margaret Raber, Jimin Wu, Hayley Donnella, Phillip Knouse, Mayurika Pise, Mark Munsell, Diane Liu, Joya Chandra

**Affiliations:** 1 Department of Pediatric Research, University of Texas MD Anderson, Houston, TX 77030, USA; MPRaber@mdanderson.org (M.R.); hjdonnella@gmail.com (H.D.); phillipknouse@outlook.com (P.K.); Mayurika.N.Pise@uth.tmc.edu (M.P.); 2 Department of Biostatistics, University of Texas MD Anderson Cancer Center, Houston, TX 77030, USA; JWu5@mdanderson.org (J.W.); mark.f.munsell@gmail.com (M.M.); dianeliu@mdanderson.org (D.L.)

**Keywords:** nutrition, oxidative stress, childhood cancer

## Abstract

Over and under nutrition are associated with worse outcomes for children with leukemia and lymphoma; however, the molecular basis for this clinical observation is not well understood. Many chemotherapeutics used for leukemia treatment are known to generate oxidative stress in vitro; therefore, we evaluated redox status and diet in pediatric leukemia patients during therapy in order to ascertain relationships between nutrition and oxidative stress. Dietary intake and redox measures in peripheral blood mononuclear cells from 32 pediatric leukemia and lymphoma patients were collected over six months during treatment. Baseline measures when patients were off chemotherapy and subsequent assessments were collected after one, two and six months. Oxidative stress increased over time in all patients, consistent with chemotherapy-induced redox effects. Older and younger children showed significantly different baseline levels of reactive oxygen species, which increased over time in all age ranges. Diet was assessed at points proximal to oxidative stress measurements and revealed a novel association with consumption of animal protein, vegetable protein, and total protein intake. Our findings demonstrate that chemotherapy increases oxidative stress in pediatric leukemia patients, and raises the possibility that dietary protein or altered protein metabolism could contribute to clinical outcomes.

## 1. Introduction

Leukemias and lymphomas are the most commonly diagnosed types of childhood cancer worldwide [1], and are responsible for the most cancer deaths among children [1]. Thus, underutilized avenues of care must be exploited to improve the survival of children with leukemia and lymphoma. Nutrition is a documented determinant of clinical outcomes for pediatric cancer patients. As many as 46% of children and young adults receiving treatment for cancer experience malnutrition [2], and several studies suggest that malnutrition portends worse outcomes for children with cancer. For example, under nutrition at diagnosis is associated with decreased 5-year survival [3] and inferior event free survival [4] of children with acute leukemia. Malnourishment at diagnosis has been associated with an increase in treatment related neutropenia in children being treated for Burkitt lymphoma [5]. Conversely, excessive body weight caused by over nutrition is associated with an increase in risk for relapse in children treated for acute lymphoblastic leukemia [6]. Overweight and obesity can also exacerbate iatrogenic effects of treatment including heart disease and osteoporosis as pediatric survivors transition into adulthood [7]. Thus, although there is strong evidence that nutrition has prognostic significance for pediatric patients with leukemia and lymphoma, few studies have attempted to clarify the molecular basis for this observation.

Cellular oxidative stress is associated with both dietary intake and cancer therapy. Oxidative stress represents a state of unbalance between reactive oxygen species (ROS) and cellular antioxidants. It has been demonstrated that dietary intake alters or modifies ROS levels in healthy populations. Meydani et al. [8] reported that restricting overweight adults’ calorie consumption for 6 months increased the plasma levels of the antioxidant enzyme glutathione peroxidase and decreased the plasma levels of protein carbonyls, which suggest that reduced calorie consumption may reduce oxidative stress. Dietary phytochemicals have also been explored as potential regulators of oxidative stress in the context of cancer prevention and treatment [9].

While oxidative stress under normal conditions may lead to the progression of disease or stress-induced DNA damage, increased levels of intracellular oxidative stress are thought to contribute to the efficacy of several chemotherapeutic regimens utilized in pediatric leukemia and lymphoma patients. These chemotherapeutic agents capitalize on the cellular redox system to increase ROS in cancer cells to the point of irreparable damage. Increased cellular oxidative stress during cancer treatment has been previously shown in pediatric patients. Papageorgiou et al. [10] demonstrated total antioxidant capacity of plasma from children with various malignancies was reduced during chemotherapy compared to capacity at diagnosis and after treatment. However, this study used total antioxidant capacity, a combination of endogenous and dietary anti-oxidants, as opposed to direct measures of oxidative stress, and did not explore factors that may influence redox.

Associations between dietary compounds and oxidative stress during chemotherapy may reveal some molecular basis for the impact of nutrition during treatment on patient outcomes. Since many chemotherapeutics used for the treatment of pediatric malignancies increase levels of reactive oxygen species (ROS) [11] and because diet may modulate the balance of cellular pro- and antioxidants, we sought to correlate the nutritional status of pediatric cancer patients receiving therapy with changes in direct measures of oxidative stress. Childhood leukemia and lymphoma patients were followed for six months during chemotherapy regimens. Oxidative stress increased over this time frame and was associated with protein consumption.

## 2. Materials and Methods

### 2.1. Participants and Procedure

The Institutional Review Board of the University of Texas MD Anderson Cancer Center approved this protocol (2009-0954). This was a prospective cohort pilot study. Eligible participants were seven years old or younger who were undergoing treatment for leukemia or lymphoma at the MD Anderson Children’s Cancer Hospital. Patients were excluded from the study if they were already enrolled on a dietary intervention. Participants were identified as potentially eligible for this study by their attending physician or study staff. Recruitment was conducted in the clinic by examining scheduled appointments weekly, and eligible patients were approached for study participation during scheduled appointments. Participation was completely voluntary, and was approved by the patient’s attending pediatric oncologist. Parents of all participants completed an informed consent or provided assent prior to data collection.

**Figure 1:**
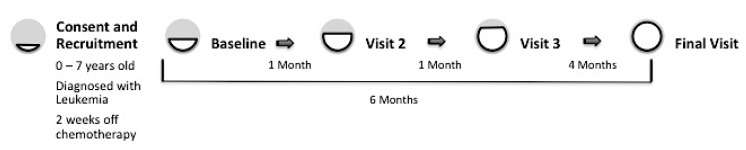
Scheme depicting participant progression through the study. After recruitment and informed consent, participants were scheduled for a baseline visit collecting demographic, diet (24 h recall), blood, and medical record information. Visits thereafter included all of these measurements except demographics. Participation time in the study was approximately 6 months from baseline to final visit. Progression through the study is represented by a gradually filling circle (from grey to white).

This study utilized peripheral blood samples to assess oxidative stress in mononuclear cells isolated from participating pediatric cancer patients. Dietary recalls were conducted at time points coinciding with blood draws. Baseline measures were captured when participants were off chemotherapy for at least two weeks but not prior to initiation of treatment, and participants were followed for six months with additional data collection at baseline, one month, two months and six months as shown in Figure 1.

### 2.2. Cells

Peripheral blood mononuclear cells (PBMCs) were isolated as follows. Five milliliters of whole blood, drawn from study participants into heparin-containing vaccutainer tubes, were slowly layered on top of an equal volume of Ficoll-PaqueTM PLUS (GE Healthcare Humble, TX, USA) and centrifuged at 1500 rpm for 30 min at room temperature. The fraction containing PBMCs was isolated, washed twice in phosphate buffered saline (PBS), and counted with a hemocytometer after staining with trypan blue. The Jurkat T-cell leukemia line was purchased from the American Type Culture Collection (Manassas, VA, USA). They were grown in a humidified incubator with 5% CO_2_ at 37 °C and were cultured in RPMI 1640 with 10% (v/v) heat-inactivated fetal bovine serum (Hyclone, Logan, UT, USA), 2 mM L-glutamine, 100 U/mL penicillin, and 100 μg/mL streptomycin (Sigma, St. Louis, MO, USA).

### 2.3. Quantification of ROS

Intracellular superoxide and hydrogen peroxide levels were measured using dihydroethidium [12] (HE) (Molecular Probes/Invitrogen, Carlsbad, CA, USA) and 5-(and-6)-chloromethyl-2′,7′-dichlorodihydrofluorescein diacetate [13], acetyl ester (DCF-DA) (Molecular Probes/Invitrogen, Carlsbad, CA, USA), respectively. Briefly, 5 × 105 PBMCs were resuspended in 1 mL PBS containing 10 µM HE or 1 mL RPMI containing 10 µM DCF and incubated for 30 min at 37 °C in the dark. Cells were then washed twice with PBS and read on the FL-1 (for hydrogen peroxide) or FL-3 (for superoxide) channel of a Becton Dickinson fluorescence-activated cell sorter (FACS Caliber; Becton-Dickinson, Franklin Lakes, NJ, USA). Jurkat cells (5 × 105) were treated with 2 mM hydrogen peroxide (Sigma, St. Louis, MO, USA) for 15 min and stained as above as a positive control for ROS staining. Results were analyzed using Cell Quest Software (Becton Dickinson). Mean fluorescence for each sample was calculated by subtracting baseline fluorescence (unstained cells) then normalizing to the cell number.

### 2.4. Quantification of Glutathione

Intracellular glutathione levels were measured [14]. Briefly, 1 × 10^6^ PBMCs were resuspended in 1 mL of a 50 mM monochlorobimane (Sigma, St. Louis, MO, USA) solution, and incubated for 15 min at 37 °C in the dark. After incubation, the stain was quenched by adding 50 µL of trichloroacetic acid to each sample and samples were centrifuged at 10,000 rpm for 5 min at room temperature. One milliliter of the supernatant was then layered on top of an equal volume of dichloromethane in a glass tube and the sample was centrifuged at 3500 rpm for 2 min at room temperature. The aqueous supernatant (200 uL/well) was plated in duplicate in an opaque 96-well plate and the fluorescence was read immediately on a Spectra GemniEM plate reader at excitation 360/40 nm and emission 460/40 nm. Standards of known concentrations of reduced glutathione were prepared in parallel with each sample and were used to calculate glutathione concentration. Jurkat cells (1 × 10^6^) were treated with 2 mM glutathione ethylester (Sigma, St. Louis, MO, USA) for 15 min or 2 mM hydrogen peroxide (Sigma, St. Louis, MO, USA) as a positive control and negative control, respectively. Results were analyzed using Microsoft Excel 2010. Mean fluorescence for each sample was calculated by subtracting baseline fluorescence (unstained cells) then normalizing it to the cell number.

### 2.5. Nutrition Measurements

Twenty-four hour dietary recalls were completed at each assessment to determine the participants’ habitual food intake. Study staff met participants’ guardians in person at the MD Anderson Children’s Cancer Hospital to complete the dietary recalls. Participants’ guardians were asked to recall the foods consumed by the participant during the previous 24 h, including details about the timing of meals, food preparation, and portion sizes. Guardians were also provided with photographs and measuring supplies to aid in the accurate recall of portion sizes when needed. The consumption of specific micronutrients was measured by analyzing the dietary intake information using the University of Minnesota Nutrition Data System for Research (NDSR) Software, version 2011.

### 2.6. Demographic and Anthropometric Measurements, and Assessment of Blood Counts

We examined participant medical records to record basic demographic information as well as changes in height and weight during the course of our study. Height and weight were recorded on the days that blood samples were collected and processed for markers of oxidative stress. Body mass index (kg/m^2^) was calculated using the height and weight. Participant medical records were also examined to determine white blood cell counts, hemoglobin levels, platelet counts, and whether patients exhibited signs of infection, such as fever. All these measures were collected at each of the visits depicted in Figure 1.

### 2.7. Response to Therapy

To measure response to therapy, we examined the participant medical records at each assessment for adverse events as defined by the Common Terminology Criteria for Adverse Events, version 3. We also used the medical records to record the presence or absence of minimal residual disease (defined as greater than 0.01% leukemia blasts in bone marrow measured by flow cytometry) and absolute lymphocyte counts at day 28 or 29, two common indicators of prognosis.

### 2.8. Statistical Analysis

Demographic information was summarized using descriptive statistics including mean, standard deviation, median, and range for continuous variables, and frequency and proportion for categorical variables. Association between categorical variables was examined by Chi-Squared test or Fisher’s exact test when appropriate. Linear regression was used to examine differential changes over time for oxidative stress measures. Oxidative stress measures were log-transformed and fitted into a linear mixed model, which included a group effect of age, time effect of visit, and interaction effect between age and visit. Dietary data were extracted from 24 h recalls and analyzed using the Nutrition Data System for Research (NDSR). Nutrition and clinical measures were added to the model separately. All computations were carried out in SAS 9.3 (SAS Institute Inc., Cary, NC, USA).

## 3. Results

### 3.1. Participants

A total of 32 childhood leukemia/lymphoma patients (aged 6 months to 7 years) completed the study protocol (Figure 1). Participant demographic and clinical characteristics are shown in Table 1; 59.4 percent of the children were male, and the median age was 3.6 year old.

Older participants were defined as those at or over 48 months of age at enrollment. Younger participants were over 6 months, but under 48 months. There were no significant differences between the older and younger groups with regard to sex, diagnosis, enrollment stage, and minimal residual disease MRD status. Participants varied by stage at which enrolled with 37.5% enrolled at induction or delayed intensification, and 62.5% enrolled at consolidation or maintenance. Study participants were grouped by age status.

### 3.2. Changes in Oxidative Stress and Diet

**Figure 2:**
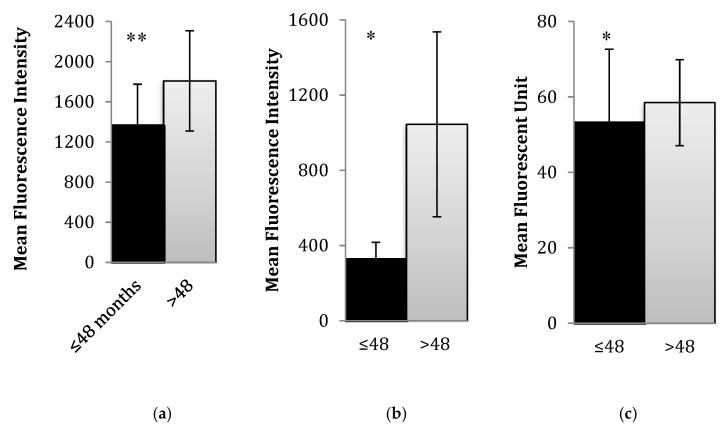
Redox differences in peripheral blood cells collected at baseline between the younger (≤48 months) and older (>48 months) group. Oxidative stress levels were significantly different between the older and younger group for all measures including (**a**) hydrogen peroxide measured by dichlorodihydrofluorescein (DCF) fluorescence, (**b**) superoxide measured by hydroethidine (HE) fluorescence, and (**c**) glutathione measured by monochlorobimane (mBCI) fluorescence. Mean fluorescent intensity (MFI) for each of these dyes is shown on the y axis of each graph. Peripheral blood samples from pediatric cancer patients and survivor participants were used. Mononuclear cells were isolated to conduct measurements of oxidative stress. Intracellular levels of free radicals were assessed using flow cytometry. Levels of the most abundant cellular antioxidants in mononuclear cells were determined using fluorometric and photometric assays. Normalized (log-transformed) data by age group and visit is shown in Table 2. Significance of associations is marked with * (*p* < 0.05) or ** (*p* < 0.01).

We sought to explore changes in both nutrition and oxidative stress among participants across the study duration in order to examine trends that occur during the course of therapy. Participants were separated into younger (younger than 4 years) and older (greater than 4 years of age) groups. This separation was made as younger children are more likely to have some intake of formula or breast milk, and caloric intake and needs would reasonably vary from older children. Significant differences in oxidative stress measures (superoxide, hydrogen peroxide and glutathione) were found between the older and younger groups at baseline (Figure 2a–c and Table 2). Levels of all oxidative stress measures were higher in the older group.

**Figure 3:**
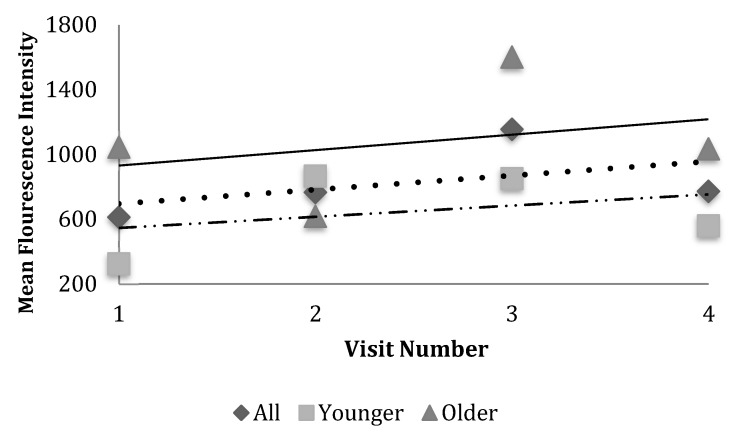
Scheme illustrating change in mean superoxide levels over visits. Peripheral blood samples assessed using flow cytometry. Superoxide, measured by HE, was different between older and younger groups. Log transformed measures were fitted into a mixed model which demonstrated superoxide level was different between older and younger groups (*p* = 0.0413), changed over time (*p* = 0.0434), and changed in the same direction over time (increase: *p* = 0.2854).

Superoxide changed significantly over time for both groups (*p* = 0.043) but did not differ significantly between groups (*p* = 0.285); although the older group appeared to have a more dramatic increase than the younger group (Figure 3, Table 2). Superoxide levels appeared to increase over the course of chemotherapy independent of participant dietary composition (Figure 3, Table 3), which remained relatively consistent over the study period.

Calorie consumption at baseline was not significantly different between the two groups; however, the older group consumed slightly more than the younger group. The macronutrient composition of the entire group was consistent in the baseline and the final visit and did not vary by age group of visit over the course of the study (Table 3).

### 3.3. Oxidative Stress Is Associated with Certain Dietary Components

Given the observed relationship between cancer prognosis and nutrition in pediatrics, and the increase in superoxide over the study period, we sought to explore the relationships between nutrition and oxidative stress. Oxidative stress measures were associated with several dietary components (Table 4).

With regard to pro-oxidants and micronutrients, hydrogen peroxide levels were significantly associated with glutamic acid (*p* = 0.044). With regard to anti-oxidants, glutathione was associated with vitamin B3 (*p* = 0.040), also known as niacin. Interestingly, both of these micronutrients are found in meat products such as beef. Total, vegetable and animal protein was associated with both hydrogen peroxide (*p* = 0.053, 0.0001, 0.0001) and superoxide (*p* = 0.0226, 0.0349, 0.0192) levels. Glutathione was associated with vegetable protein (*p* = 0.029). The dietary components found to be significant in Table 4 were added to a mixed model adjusted for age and visit (Table 5). This model confirmed a strong association between hydrogen peroxide and total protein (*p* = 0.0002) and riboflavin (*p* = 0.044), also known as vitamin B2, which is found in dairy and meat products. Glutathione and vegetable protein also continued to show an association in the revised model (*p* = 0.029).

### 3.4. Oxidative Stress and Clinical Characteristics

Certain clinical characteristics are also associated with nutrition and may offer further insight into the observed relationship between oxidative stress measures and diet. Therefore, we examined associations between oxidative stress measures and clinical characteristics such as anthropometrics, white blood cell (WBC) counts, hemoglobin (Hb), platelets, and infections (Table 6).

Hydrogen peroxide was significantly associated with weight (*p* = 0.0497) and platelet count (*p* = 0.004), but not BMI (*p* = 0.6846). Superoxide was significantly associated with height (*p* = 0.0436). Our data suggested no relationship between oxidative stress and infection. Less than 22% of participants had infections during any visit; most infections were low grade (grade 1 or 2) and there was only one incidence of higher grade infection (grade 4) throughout the study period.

## 4. Discussion

This pilot study explored the relationship between nutrition, therapy response and redox status in 32 pediatric leukemia and lymphoma patients over a six-month window of chemotherapy. We demonstrated that intracellular superoxide, peroxide and glutathione levels increased over time in the six-month period of chemotherapy treatment for the entire sample and were associated with animal protein, vegetable protein, and total protein intake. Younger children (under 48 months) and older children (over 48 months) showed significantly different baseline levels of reactive oxygen species, a measure of oxidative stress. This difference in younger versus older participants may be attributable to lower mutation rates, less exposure to environmental carcinogens, age associated epigenetic differences, or cell cycle rates, and was not addressed in our current investigations but is worthy of future study.

Changes in hydrogen peroxide and superoxide showed a positive trend in the older group, but not the younger group. This change appeared to be independent of changes in macronutrient intake as participant diet did not shift dramatically over the study period (Table 3). These findings support previous observations that certain chemotherapeutic drugs may function through increased generation of ROS over the course of active treatment [15,16]. It is unclear why trends differed by age group; this finding warrants further exploration in future studies.

Glutathione was positively associated with vegetable protein. Glutathione is an abundant antioxidant with the capacity to counter oxidative stress damage within cells. Elevated glutathione levels has been positively associated with relapse of disease among pediatric ALL patients [17]. Given that many chemotherapeutics used in ALL therapy optimize the oxidative stress pathway to cause cancer cell death, a deeper examination of the impact of diet on glutathione levels during treatment is warranted.

This finding is in line with previous research that showed differentiation between animal and vegetable protein with regard to ROS. Gu et al. found that high casein protein (derived from dairy) diets increased ROS in duodenum, liver, and pancreas of mice. However, this increase was not found in mice fed high soy protein diets [18]. Although different ROS measures were used in this study than those described in the current work, these findings suggest vegetable as opposed to dairy protein may have different impacts on ROS generation, potentially due to glutathione’s ability to remove ROS from leukemia cells [17].

The interplay of dietary protein sources, pro-oxidant generation, anti-oxidant generation, and cancer prognosis is still unclear. More recent work using the Eμ-Myc-lymphoma mouse demonstrated that an overall low protein diet significantly slowed cancer progression [19]. These findings highlight the need for more nuanced research examining the mechanisms linking diet, oxidative stress, and cancer. Prospective applications of this research may include investigating the modulation of protein consumption concomitant with chemotherapy in this population. Detailed investigation into the role of specific amino acids or specific types of protein sources in augmenting chemotherapy hold potential to improve outcomes for leukemia and lymphoma patients through diet modification.

This work is unique as it examines direct measures of oxidative stress, as opposed to more commonly used measures of overall antioxidant capacity, thus, deepening our understanding of the role specific antioxidants play in redox homeostasis during therapy. Our findings demonstrate proof of principle that chemotherapy drugs increase direct measures of oxidative stress, and identify a novel association of protein intake with oxidative stress measures in pediatric tumor patients.

This study has several limitations including a relatively small sample size, which limits the generalizability of our results. The sample population was inclusive of a range of different pediatric hematological malignancies (Table 1) and enrollment occurred at different stages of treatment. Thus, we cannot determine if all chemotherapeutic drugs impacted oxidative stress measures or only specific ones. Some of most commonly used drugs in our sample included vincristine, methotrexate, and dexamethasone. Dexamethasone is known to influence appetite and was not consistently delivered or dosed across all patients studied because of the range of diagnoses and stages. Glutathione and oxidative stress levels may impacted by transfusions and infection. Infection status was not associated with oxidative stress in our study (Table 6); however, transfusion status was not examined. Finally, dietary recalls may be subject to recall bias and may not accurately capture a patient’s true diet. No data on nutrient absorption or data on alternative modulators of vitamin levels (i.e., vitamin D from sunlight) were collected, and this is an important variable in considering biological effects. Future research should consider examining oxidative stress relative to dietary intake variables among larger and more diverse patient populations.

## Figures and Tables

**Table 1:** Summary of Demographic and Clinical Characteristics of Sample by Age Group.

		Total n (%)	Age		*p*-Value
			≤48 Months	>48 Months	
Sex	Female	13 (40.6%)	8 (42.1%)	5 (38.5%)	1.0000
	Male	19 (59.4%)	11 (57.9%)	8 (61.5%)	
Diagnosis	Acute Myeloid Leukemia (AML)	2 (6.3%)	2 (10.5%)	0 (0%)	0.1238
	Anaplastic Large cell Lymphoma	1 (3.1%)	1 (5.3%)	0 (0%)	
	High Risk pre-B Acute Lymphocytic (or lymphoblastic) Leukemia (ALL)	8 (25%)	5 (26.3%)	3 (23.1%)	
	Relapse ALL	3 (9.4%)	0 (0%)	3 (23.1%)	
	Standard Risk pre-B ALL	14 (43.8%)	8 (42.1%)	6 (46.2%)	
	T-cell ALL	3 (9.4%)	3 (15.8%)	0 (0%)	
	T-cell Lymphoma	1 (3.1%)	0 (0%)	1 (7.7%)	

**Table 2:** Oxidative Stress Measures Over Time by Age Group and Visit.

Log (Ox Stress Measure)	Age	Visit	N	Mean	S.D.	Mixed Model Effect (*p*-Value)		
						Age	Visit	Age × Visit
Hydrogen Peroxide	≤48 M	1	19	6.33	1.60	0.007	0.464	0.798
		2	19	6.19	1.56			
		3	19	6.58	1.08			
		4	16	6.59	1.23			
	>48 M	1	12	7.08	0.98			
		2	13	7.02	1.43			
		3	13	7.33	1.35			
		4	12	7.85	1.13			
Superoxide	≤48 M	1	18	5.04	1.42	0.041	0.043	0.285
		2	19	5.23	1.56			
		3	19	5.94	1.33			
		4	16	5.39	1.42			
	>48 M	1	12	6.19	1.35			
		2	13	5.49	1.87			
		3	13	6.31	1.67			
		4	13	6.61	0.79			
Glutathione	≤48 M	1	18	3.36	1.07	0.016	0.333	0.355
		2	19	3.36	1.19			
		3	18	3.47	1.10			
		4	16	3.29	1.22			
	>48 M	1	13	3.83	0.74			
		2	13	4.24	1.07			
		3	13	4.26	1.22			
		4	12	4.51	0.71			

Oxidative stress measures were fitted into a mixed model, which included a group effect of age, time effect of visit, and interaction effect between age and visit. N = number of patients assessed, M = months, S.D. = standard deviation. A significant age group effect meant a significantly difference in mean intercepts of outcomes between age groups. A significant visit effect meant that the outcome changed over time.

**Table 3:** Kilocalorie and macronutrient intake by age group.

	Visit 1 Mean (Range)			Visit 4 Mean (Range)		
	≤48	>48	All	≤48	>48	All
Protein (g)	52	68	58	44	60	51
	(15–166)	(22–129)	(15–166)	(19–70)	(31–104)	(19–104)
Fat (g)	59	61	59	48	58	53
	(20–197)	(14–118)	(13–197)	(11–86)	(22–97)	(11–97)
Carb (g)	203	225	212	189	216	201
	(85–488)	(80–605)	(80–605)	(67–453)	(96–300)	(67–453)
Kilocalories	1523	1687	1590	1366	1560	1453
	(720–3182)	(672–3934)	(672–3934)	(444–2284)	(707–2326)	(444–2326)

**Table 4:** Multi-level regression model examining the relationships between oxidative stress measures ^a^ and dietary components.

	Association (*p* Value)		
Dietary Components	Hydrogen Peroxide (DCF)	Superoxide (HE)	Glutathione (mBCI)
Kilocalories	0.1904	0.4873	0.2428
Riboflavin (mg)	0.0314 *	0.6332	0.4172
Combined Carotene	0.0845	0.1455	0.8918
Iron (mg)	0.2496	0.753	0.9812
Vitamin A (IU)	0.9154	0.2002	0.2876
Vitamin B3 (mg)	0.8738	0.2538	0.0401 *
Vitamin C (mg)	0.3989	0.9296	0.4837
Vitamin E (IU)	0.1719	0.8186	0.7604
Glutamic Acid (g)	0.0444 *	0.2042	0.0646
Selenium (mcg)	0.4216	0.076	0.5178
Vitamin D (µg)	0.841	0.7973	0.6851
Natural Vitamin E (mg)	0.4118	0.3922	0.5751
Synthetic Vitamin E (mg)	0.6466	0.7128	0.2702
Total Protein (g)	0.0534 *	0.0226 *	0.719
Animal Protein (g)	0.0001 **	0.0192 *	0.6254
Vegetable Protein (g)	0.0001 **	0.0349 *	0.0286 *

Significance of positive associations is marked with * (*p* < 0.05) or ** (*p* < 0.01). ^a^ Oxidative stress measures are log transformed.

**Table 5:** Multi-level regression model adjusted for age and visit examining the relationships between oxidative stress measures ^a^ and dietary components.

	Association (*p* Value)		
Dietary components	Hydrogen Peroxide (DCF)	Superoxide (HE)	Glutathione (mBCI)
Riboflavin (mg)	0.0444 *	NS	NS
Combined Carotene (mcg)	0.0820	NS	NS
Total Protein (g)	0.0002 **	NS	NS
Selenium (mcg)	NS	0.0760	NS
Vegetable Protein (g)	NS	NS	0.0286 *

Significance of positive associations is marked with * (*p* < 0.05) or ** (*p* < 0.01). ^a^ Oxidative stress measures are log transformed.

**Table 6:** Multi-level regression model examining the relationships between oxidative stress measures ^a^ and clinical characteristics.

	Association (*p* Value)		
Clinical Characteristics	Hydrogen Peroxide (DCF)	Superoxide (HE)	Glutathione (mBCI)
Height (cm)	0.1084	0.0436 *	0.9852
Weight (kg)	0.0497 *	0.1119	0.9119
BMI (kg/m^2^)	0.6846	0.4628	0.2736
WBC Count (K/µL)	0.0765	0.5209	0.9234
Hb (G/dL)	0.4157	0.1066	0.9386
Platelets (K/µL)	0.004 **	0.1327	0.3905
Any Infections (Y/N)	0.1465	0.9638	0.7497

Significance is marked with * (*p* < 0.05) or ** (*p* < 0.01). ^a^ Oxidative stress measures are log transformed.
